# Sudden infant death syndrome revisited: serotonin transporter gene, polymorphisms and promoter methylation

**DOI:** 10.1038/s41390-021-01773-3

**Published:** 2021-11-11

**Authors:** Nina Pfisterer, Fiona Meyer-Bockenkamp, Dong Qu, Vanessa Preuss, Thomas Rothämel, Dorothee Geisenberger, Katharina Läer, Benedikt Vennemann, Anne Albers, Theresa A. Engelmann, Helge Frieling, Mathias Rhein, Michael Klintschar

**Affiliations:** 1 Department of Psychiatry, Social Psychiatry and Psychotherapy, Laboratory for Molecular Neuroscience, Hannover Medical School, Feodor-Lynen-Str. 35, 30625 Hannover, Germany; 2 Institute of Forensic Medicine, Hannover Medical School, Carl-Neuberg-Str. 1, 30625 Hannover, Germany; 3 Institute of Forensic Medicine, University of Freiburg, Faculty of Medicine, Freiburg, Germany

## Abstract

**Background:**

Based on findings in the brain stems of SIDS victims, the serotonin transporter (5-HTT) gene has been discussed to be associated with SIDS.

**Methods:**

In the largest study to date, we investigated the promoter length (5-HTTLPR) and intron 2 VNTR polymorphisms in 274 cases and 264 controls and the Ile425Val polymorphism in 65 cases and 64 controls. Moreover, the methylation of the internal promoter region was investigated in 35 cases and 14 controls.

**Results:**

For 5-HTTLPR, we observed a trend towards an association of allele L (58.8% vs. 53.4%) with SIDS and significant results were observed after stratifying for age, season at death, and prone position. Nevertheless, when pooling all published data, a significant association of allele L with SIDS is confirmed (*p*: 0.001). For the intron 2 VNTR polymorphism, no significant differences were observed. After pooling, a significant accumulation of the rare allele 9 was observed in SIDS (2.1% vs. 0.6%; *p*: 0.018). For the Ile425Val polymorphism, no differences were observed.

**Conclusion:**

We conclude that genetic variation at this gene might be of some importance in SIDS. Epigenetic analysis of the internal promoter, however, revealed no influence on the relative risk to succumb to SIDS.

**Impact:**

- This is the largest study published up to now on 5-HTT gene polymorphisms and SIDS.
- Polymorphisms in the 5-HTT gene appear to contribute (although to a small degree) to the risk to die from SIDS.
- There is no evidence that a methylation of the promoter region is of impact for the etiology of SIDS.

## Introduction

Sudden infant death syndrome (SIDS) is the leading cause of death in infants older than 1 week of age^1^ and its etiology is still largely obscure^2,3^. The triple risk theory^4^ involves the intersection of (i) a vulnerable infant, (ii) a critical developmental period, and (iii) exogenous stressors as cause for SIDS. The vulnerability is attributed mainly to an impaired autonomic nervous system, most notably concerning the respiratory regulation and/or arousal reaction^5^. Serotonergic neurons constitute a major modulating system that controls the respiratory activity and these functions are adversely affected as demonstrated by numerous reports on multiple abnormalities in the serotonergic system of the brain stem^6–8^. Furthermore, the influence of genetic variants is discussed: several studies have been published on gene variants of relevance for the serotonergic system and other parts of the autonomous nervous system^9–12^.

The most prominent gene in that respect is the serotonin transporter (5-HTT) gene that is responsible for the reuptake of released serotonin (5-HT) and thus regulates the level of 5-HT in the synaptic cleft^13^. A promoter polymorphism of the 5-HTT gene (5-HTTLPR) alters the transcription rate of the gene: a promoter with the short “S” allele (14 copies of a 22–23 bp repeat region) is less active in vitro compared to the longer “L” allele (16 copies)^14,15^. After an initial description of an increased frequency of L-alleles in a small Japanese SIDS group^16^, Weese–Meyer corroborated this finding in Caucasians and Afro-Americans and found another 5-HTT gene polymorphism, a variable number of tandem repeat (VNTR) polymorphism in intron 2, observing an association of allele 12 with SIDS^17,18^. While the latter finding could not be confirmed by other studies, up to now the 5-HTTLPR was found to be associated with SIDS in an Italian and a Norwegian sample^19,20^ but not in a Swiss sample^21^. Even more so, Paterson et al.^22^ failed to demonstrate such an association in a sample set from the San Diego Medical Examiner’s Office (the “San Diego dataset”). The authors proposed that, at best, the 5-HTTLPR genotype is only a weak determinator of the risk to succumb to SIDS^23^. They further propose that studies of appropriate size and ethnically matched case and control datasets need to be performed before finally evaluating the role of 5-HTT gene variation in SIDS.

However, the activity of a gene is not determined by sequence variance alone. Epigenetics play an important role in developmental concerns and differentiation as well as various diseases^24^. Furthermore, for the 5-HTT gene an epigenetic effect was demonstrated for many, mostly psychiatric, conditions^25^.

CpG sites are regions of DNA where a cytosine nucleotide is followed by a guanine nucleotide. Genomic regions that are characterized by a high frequency of CpGs are referred to as CpG islands and are common in promoter regions, especially close to the transcriptional start site. The degree of methylation of these DNA stretches has an important regulatory function^24^. Even more so, it is known that age has a great impact on DNA methylation insofar as it increases with age^26^. The infant has to adapt to the new environment after birth and grows rapidly during the first year with numerous metabolic challenges. It can be assumed that these adaptation processes are at least in part mediated by epigenetic changes. Therefore, the critical developmental period postulated by the triple risk model might well be the consequence of an inadequate epigenetic regulation of genes. Thus, we hypothesized, that the 5-HTT gene might have an impact on the etiology of SIDS not so much by genetic variants but by the degree of methylation.

To test this hypothesis we genotyped the 5-HTTLPR, the intron 2 VNTR polymorphism, and a rare gene variant which leads to gain of function (Ile425Val)^27^ in the currently largest sample tested. Also, we used bisulfite sequencing to identify the methylation status of the immediate proximal promoter of the serotonin transporter gene to review its suitability as a biomarker.

## Methods

### Typing of 5-HTT polymorphisms

#### Subjects

The subjects used for this part of the study were 274 Caucasoid infants from Germany whose death occurring during sleep remained unexplained after a thorough investigation, including a complete autopsy, the review of the circumstances of death, and the clinical history, as demanded by Krous et al.^1^ for establishing the diagnosis “SIDS”. 175 of the infants were males and 99 females. The age range was between the 5 and 360 days (median 110 days). For the Ile428Val study of those cases, 65 cases were selected (age range 28–292 days, median 122 days). For most cases, information concerning the age at death and the date of death was available; for a minority, also information concerning the position in infants at death were available (40 SIDS victims were found in a prone position, for the remaining either no information was documented or they were found in another position). Only for some of the cases information regarding maturity at birth was available, wherefore postnatal age rather than postconceptional age was used for further analysis.

The controls consisted of 264 healthy young adult Caucasoid persons who had survived the critical time span of the first year without SIDS (110 females, 154 males). Sixty-four of these controls were included in the Ile428Val study. Due to anonymization, no data on age are available. The study was approved by the local ethics committee.

For the methylation study, 35 SIDS cases (age range 18–305 days, median 94 days, 21 males, 14 females) and 14 controls (age range 5–680 days, median 111 days, 8 males, 6 females) were included. As (due to the rarity of other causes of death prevalence of SIDS as cause of death) control cases are rare, we decided to include also children for up to the end of the second year. The control cases died from heart defect (4), shaken impact syndrome (4), infectious disease (4), ischemia of the colon (1), and an inborn metabolic defect (1). Three types of tissue were investigated (blood, pons cerebri, and medulla oblongata), but (due to missing samples) for pons only 24 cases and 7 controls and for the medulla only 21 cases and 6 controls could be included.

#### DNA analysis

Genomic DNA was extracted from 0.5 mL aliquots of blood samples or tissue samples, as described previously^28^.

Each 25 µL assay contained 2.5 μL 10× PCR buffer (including 15 mM MgCl_2_), 0.5 μL dNTP mix (300 μM final concentration, 0.3 μM of each primer, and 0.2 μL Taq Gold Polymerase (Life Technologies, Darmstadt, Germany).

The 5-HTTLPR polymorphism was genotyped using primers as described^16^ (forward: 5′-TCC TCC GCT TTG GCG CCT CTT CC-3′; reverse: 5′-TGG GGG TTG CAG GGG AGA TCC TG-3′). After an initial denaturation step (95 °C for 15 min), thermocycling was performed for 37 cycles of 94 °C/30 s, 65 °C/60 s and 72 °C/60 s, followed by a final extension period of 72 °C for 10 min using a FlexCycler (Analytik Jena, Jena, Germany) resulting in fragments of 484 bp (S) and 528 bp (L). These fragments were separated and detected on 2% agarose gels in the presence of ethidium bromide and visualized under UV light.

For the intron 2 VNTR polymorphism, primer sequences used were forward: 5′-TCC TCC GCT TTG GCG CCT CTT CC-3′ and reverse: 5′-TGG GGG TTG CAG GGG AGA TCC TG-3′^18^. The forward primer was labeled using the chromophor 6-FAM.

After initial denaturation (95 °C for 11 min), thermocycling was performed for 37 cycles of 94 °C/30 s, 58 °C/30 s, and 72 °C/90 s, and a final extension period of 72 °C for 10 min. Genotyping was performed on a 310 Genetic Analyzer (Life Technologies, Darmstadt, Germany) as described^29^.

The Ile425Val single-nucleotide polymorphism (SNP) was amplified using the following primers: forward: 5′-TGG AAG CCC CAC CCT TCC TG-3′ and reverse: 5′-CAT CCT CCC ACA GCC CAT TTC C-3′ ^30^ (95 °C for 11 min), 37 cycles of 94 °C/30 s, 58 °C/30 s, and 72 °C/90 s, followed by a final extension period of 72 °C for 10 min. After restriction with BccI for 2.5 h at 37 °C (New England Biolabs, Frankfurt, Germany). Samples were typed on a QIAxcel electrophoresis unit using the QIAxcel DNA Screening Kit (Qiagen, Hilden, Germany) as previously described^31^. Fragment lengths were 365 bp (unrestricted) and 288/77 bp (restricted).

### Methylation analyses

For each individual, up to three tissue samples (blood, medulla oblongata, and pons cerebri) were analyzed. DNA was extracted from tissue saved during the autopsies using the QIAamp DNA Mini Kit (Qiagen, Hilden, Germany) for blood samples on filter paper and the NucleoMag Blood 200 μL Kit (Macherey-Nagel, Düren, Germany) for deep frozen tissue samples. DNA concentration was determined using the Qubit dsDNA HS Assay Kit (Invitrogen, Carlsbad, CA).

DNA samples were bisulfite-converted and purified using the EpiTect 96 Bisulfite Kit (Qiagen, Hilden, Germany) as previously described^32^.

Primers to amplify the range 28563061–28563299 of the internal promoter region were purchased from Metabion international, Planegg/Steinkirchen, Germany (812_5HTTE-Fw-N (GAATTGTAGTTGGTTAATAAAATGAG; forward) and 814_5HTT-Rv-NI (AAAAAAACTACACAAAAAAACAAATATAC; reverse)). For sequencing, the primer 753_5HTTE-Fw2 (GAGAGGAATTAGATAAGGGTTTTTA) was used.

PCR targeting *SLC6A4* was performed using an extension temperature of 58.5 °C for 35 cycles and the purified product sequenced on a Genetic Analyzer 3500 XL (Applied Biosystems, Foster City, CA) after applying POP3.1 ddNTP chemistry for the sequencing PCR (ABI Life Technologies, Grand Island).

### Statistical analyses

Exact tests for deviation from Hardy–Weinberg equilibrium were performed using the Genepop software (http://wbiomed.curtin.edu.au/genepop/). The algorithm of Roff and Bentzen^33^ was applied to test differences in the allele frequencies between controls and SIDS cases in male and female individuals and genotype frequencies in female individuals using the RXC.exe software (George Carmody, Carleton University, Canada). Statistical significance was calculated by performing 10,000 simulations on the overall observed two-way contingency tables. The null hypothesis was rejected at a level of >0.05.

Analysis of CpG-specific methylation percentage was performed using the Epigenetic Sequencing Methylation (ESME) analysis software^34^ and the methylation rate (%) of each CpG site within the amplified region was estimated by the ratio between normalized peak values of cytosine (C) and thymine (T) as described^32^.

## Results

### All investigated loci were in Hardy–Weinberg equilibrium

Allele frequencies and genotype frequencies of the 5-HTTLPR polymorphism in SIDS and control subjects are summarized in Table 1 (left). For 273 SIDS cases and 277 controls, results were obtained. In accordance with the findings of Paterson et al.^22^ the frequency of the L-allele in Caucasian controls was well below 50% and the frequency of this allele showed a non-significant trend towards association with SIDS. Pooling data for Caucasoid populations from all previously published studies^17,19–22^ resulted in a SIDS group of 438 cases vs. a control group of 485 individuals. For these pooled groups, a strong association of allele L with SIDS was demonstrated (Table 1, right).

**Table 1:** 5-HTTLPR alleles in SIDS and controls in this study (left), pooled results for this study and all studies published up to now in Caucasians (17,19,20,21,22) (right).

	SIDS		Controls			SIDS		Controls	
Allele	*n*	%	*n*	%	Allele	*n*	%	*n*	%
S	227	41.58%	258	46.57%	S	370	42.20%	477	49.20%
L	319	58.42%	296	53.43%	L	506	57.80%	493	50.80%
*p*		n.s.			*p*		0.001	± 0.0010	
S/S	51	18.68%	71	25.63%	S/S	83	18.90%	125	25.80%
L/S	125	45.79%	116	41.88%	L/S	204	46.60%	228	47.00%
L/L	97	35.53%	90	32.49%	L/L	151	34.50%	132	27.20%
Total	273		277		Total	438		485	
*p*		n.s.			*p*		0.014	± 0.0037	

*n.s.* not significant.

Results for the intron 2 VNTR are displayed in Table 3. For 240 cases and 264 controls, results were obtained. As in previous studies, three different alleles with 9, 11, and 12 repeats were detected in a total of 504 individuals. As expected, allele 9 was the rarest of these alleles, and we observed it almost twice as frequently in SIDS compared to controls, although this difference was not significant (Table 2, left). There are only two other studies that genotyped this polymorphism and reported the results for allele 9 (refs. ^19,21^). Pooling these data (405 SIDS cases and 472 controls) yielded a statistically significant accumulation of allele 9 in SIDS (Table 2).

**Table 2:** 5-HTT intron 2 VNTR alleles in SIDS and controls in this study (left), compared to pooled results for this study and all studies published up to now in Caucasians that presented explicit allele frequencies^19^^,^^21^ (middle).

	SIDS		Controls			SIDS		Controls			SIDS		Controls	
Allele	*n*	%	*n*	%	Allele	*n*	%	*n*	%	Allele	*n*	%	*n*	%
9	9	1.88%	5	0.95%	9	17	2.10%	6	0.64%	9	17	2.10%	6	0.60%
10	195	40.63%	207	39.20%	10	317	39.14%	361	38.24%	10 + 12	793	97.90%	938	99.40%
12	276	57.50%	316	59.85%	12	476	58.77%	577	61.12%					
*p*		n.s.			*p*		0.024	± 0.0048		*p*		0.007		
9/9	1	0.42%	0	0.00%	9/9	1	0.25%	0	0.00%	9/9	1	0.10%	0	0.00%
9/10	5	2.08%	4	1.52%	9/10	7	1.73%	4	0.85%	9/10+	15	2.40%	6	1.30%
9/12	2	0.83%	1	0.38%	9/12	8	1.98%	2	0.42%	10+/10+	389	97.50%	466	98.70%
10/10	47	19.58%	43	16.29%	10/10	68	16.79%	76	16.10%					
10/12	96	40.00%	107	40.53%	10/12	174	42.96%	195	41.31%					
12/12	89	37.08%	109	41.29%	12/12	147	36.30%	195	41.31%					
Total	240		264		Total	405		472		Total	405		472	
*p*		n.s.			*p*		n.s.			*p*		0.018	± 0.003	

At the right side, the results for the pooled data are compared for allele 9 vs. all other alleles combined. *n.s.* not significant.

After stratifying our data for the 5-HTTLPR polymorphism according to sex, age, season, and position at death, these subgroups were compared to the controls. We could demonstrate a significant association with allele L for those SIDS cases that died at 46–150 days (*p*: 0.045) and those that died in a prone position (*p:* 0.011) (Table 3, left).

**Table 3:** Results for 5-HTTLPR and 5-HTT intron 2 VNTR polymorphisms in SIDS cases after stratification according to age at death, sex, season, and sleeping position compared to living controls.

	5-HTTLPR			5-HTT intron 2 VNTR			
	S	L	*P* value	9	10	12	*P* value
**Age (days)**							
0–45	39	39	0.627	1	36	41	0.471
46–150	87	139	**0.045**	2	78	146	0.470
151–365	74	102	0.336	6	81	89	**0.014**
**Sex**							
m	124	186	0.071	9	128	173	0.073
f	76	94	0.723		67	103	0.444
**Season**							
Spring	43	61	0.334	2	45	57	0.477
Summer	35	45	0.718	2	27	51	0.338
Autumn	43	57	0.584	2	51	47	**0.048**
Winter	43	65	0.205	1	40	67	0.914
**Position**							
Prone	25	55	**0.011**	2	39	39	0.106

Bold values indicate statistical significance *p* < 0.05.

For the intron 2 VNTR polymorphism, stratifying our data according to sex, age, season, and position at death demonstrated a significant association for those SIDS cases that died at 151–365 days (*p*: 0.014) and those that died during autumn (*p*: 0.048), in both cases showing a predominance of alleles 9 and 10 compared to allele 12 (Table 3).

When genotyping the Ile425Val SNP, we failed to demonstrate the 425Val variant in 129 cases (data not shown). We thus chose not to include further samples into the investigation.

A representative electropherogram for the sequencing of the immediate promoter region after bisulfite treatment is shown in Supplementary Fig. 1. The 13 CpGs in the amplified fragment all showed invariably a lack of methylation as 100% of the DNA was converted. This was demonstrated in all tissues and all samples of both SIDS cases and controls.

## Discussion

Despite the fact that no Mendelian mode of inheritance was demonstrated for SIDS, it is assumed that multiple genetic influences contribute to the risk to succumb to this syndrome^3^. Thus, various gene variants have been investigated in SIDS, among which the 5-HTTLPR polymorphism was the first polymorphism of relevance for the serotonergic system to be associated with SIDS^35^. However, as some studies failed to reproduce this effect and others showed only a weak association, it is still unclear if, and if so to what extent, this polymorphism is indeed associated with SIDS. In that respect, the 5-HTTLPR polymorphism is similar to the *MAOA* promoter polymorphism, another polymorphism of relevance for the serotonergic system that was associated with SIDS, for which also widely discrepant studies were published^11,36–38^. However, a recently published meta-analysis for this polymorphism results in an association with SIDS for this polymorphism^39^.

For 5-HTTLPR, Paterson et al.^22^ argue that, at best, it is only a weak determinator of the risk to succumb to SIDS. They demonstrate that all studies which up to now succeeded to demonstrate a significant association with SIDS showed an extremely high frequency of allele S in the controls and speculate that this association might be an artifact. However, it is evident that also the study by Paterson et al. shows a trend towards association of allele L with SIDS. In that respect, their results are markedly similar to ours. Moreover, it is worth to be noted that all published data except the study by Haas et al.^21^ showed a higher frequency of allele L in SIDS. To further evaluate this polymorphism we pooled the data from five previously published studies on Caucasians^17,19–22^ with our new data (Table 1, center) and found under these conditions a strongly significant association with SIDS. We therefore consider it likely that 5-HTTLPR indeed is associated with SIDS and that this polymorphism is involved in the etiology of this syndrome. Nevertheless, we agree with Paterson et al. that this association most likely is weak, especially when comparing it to the effect of other risk factors, most notably the sleeping position.

The intron 2 VNTR polymorphism showed only significance in one of four published studies, and our study also failed to demonstrate a significant association (Table 2, left). However, we observed the rare allele 9 slightly more often in the SIDS group compared to the control group (1.88% vs. 0.95%). While this finding was not significant in our sample, we succeeded to demonstrate significance after pooling our results with the two studies found in the literature that unambiguously displayed all alleles^19,21^. The study of Weese-Meyer et al.^18^ could not be included as this study pooled the rare allele 9 with the more common allele 10 and the study by Opdal et al.^20^ was excluded as it was not possible to retrieve the exact data from the figure that displayed the results. At any rate, the originally described association of allele 12 with SIDS in Afro-Americans could not be demonstrated in the Caucasian studies included in this analysis. Allele 9 was reported to have a strong positive influence on the expression of the 5-HTT gene *SLC6A4* (ref. ^40^). Therefore, as for the L-allele of the 5-HTTLPR polymorphism, the association of allele 9 with SIDS would lead to lower synaptic serotonin levels, a finding observed for instance by Panigrahy et al.^41^, and thus supporting the brainstem hypothesis of SIDS^5^.

The Ile425Val SNP shows a very rare functional variant, 425Val. We therefore speculated that it might be involved in the etiology of SIDS. Yet, we failed to demonstrate this variant in both controls and SIDS cases and thus conclude that this polymorphism is probably of no interest in SIDS.

We performed the first methylation study on the promoter region of the 5-HTT gene in association with SIDS. In fact, to the best of our knowledge, to date only one methylation study was published on SIDS cases, targeting a CpG island located upstream of the promoter of the growth factor independent 1 (GFI1) gene depending on the fact whether the parents of the deceased infant were smokers or not^42^.

Methylation adapts the genome to changing requirements due to diseases, chronic intoxications, ageing, and even traumatic life events^43^. The first year of life is a time of constant adaptation to changing needs. On these grounds, one of the theoretic approaches to SIDS is that the infant is maladapted to the current environmental and biological conditions. Differences in DNA methylation might very well explain this maladaptation. It is therefore somewhat surprising that the epigenetics of SIDS have been largely neglected up to now. In our study all children, controls, as well as SIDS cases lacked any methylation in the internal promoter region. Although the lack of methylation reported herein does not support a potential epigenetic influence in the etiology of SIDS, we propose to extend epigenetic studies to other genes that might be of relevance for current concepts in SIDS, i.e. the triple risk hypothesis, and of which some have already been, with rather ambiguous results, been associated to SIDS on the genomic level.

Age and season at death are external factors of high importance in SIDS, with peak SIDS prevalences between the second and fourth month of life and during the cold months of the year, respectively. Also, boys are at an approximately 50% higher risk than girls. The most influential factor determining the SIDS risk, however, is a prone sleeping position. As SIDS is a multifactorial syndrome (comprising deaths from arrhythmias, metabolic, immunologic, or respiratory derailment), we speculate that the presence or absence of one of these factors could help to further categorize Sudden infant death: Infants dying in a prone position, for instance, might more often die from brain stem- and serotonin-related respiratory failure, whereas children found in a supine position might show a preponderance of other causes. For example, in an earlier study, we succeeded to demonstrate an association with the age of 46–150 days for the THO1 locus^12^. We therefore stratified our data for the SIDS group according to these factors and compared these groups to the control group. For age, sex, and season this information was available for almost all samples. However, only for 40 deceased infants it was documented that those were found in a prone position. The subgroups were compared to the controls rather than the remaining SIDS samples.

Most interestingly, the conditions with the highest prevalence of SIDS (age 46–150 days and prone position) are those with a significant correlation to allele L of 5-HTTLPR (Table 3). This finding appears to support the notion that the subtype of SIDS appears to actually vary depending on the age and sleeping position at death.

However, it must be noted that Opdal et al.^20^ found an association between S/S genotype and prone position—not the L-allele as in our study. This finding emphasizes that size is a crucial quality in association studies and that detailed conclusions are only possible after a sufficient number of sufficiently large studies (our study could include only 40 infants with known prone position, that of Opdal and colleagues comprised 93 infants with prone position). Future studies should focus on the position in which the child was found.

Further, we found a very weak association for intron 2 VNTR with autumn and a stronger one with death during days 151–365 of life. However, in both cases not the initially reported allele 12, but the alleles 9 and 10 did predominate. While we consider the association with autumn most likely a statistical artifact, the association of this locus with older children seems to be more plausible, and one explanation might be that both loci have different impact on the serotonergic system.

In conclusion, we succeeded to demonstrate significant associations of two polymorphisms studied herein (5-HTTLPR and intron 2 VNTR) with SIDS, albeit only after stratification according to position, age, and season at death. We argue that stratification of SIDS samples might be a valuable approach in studies concerning the genetic background of SIDS, as it could enable the researcher to reduce the bias caused by the diverse etiology of SIDS. Moreover, when pooling our results with those found in the literature, we found significant associations for both polymorphisms. Meta-analytic studies are a method to increase the power of an association study but are undoubtedly limited and prone to inherent problems. Yet, our results suggest that both polymorphisms indeed contribute to the risk of dying suddenly during the first year of life, but that the risk conferred by these alleles is relatively small, especially when compared to that conferred by some external risk factors like, e.g., the prone vs. supine sleeping position.

## Supplementary information

Supplementary Material
