# Supplementary figures and images for: Sudden infant death syndrome revisited: serotonin transporter gene, polymorphisms and promoter methylation
Source: Pediatr Res. 2021 Nov 11;92(3):694–9. doi: 10.1038/s41390-021-01773-3 (PMC9556327; doi:10.1038/s41390-021-01773-3)

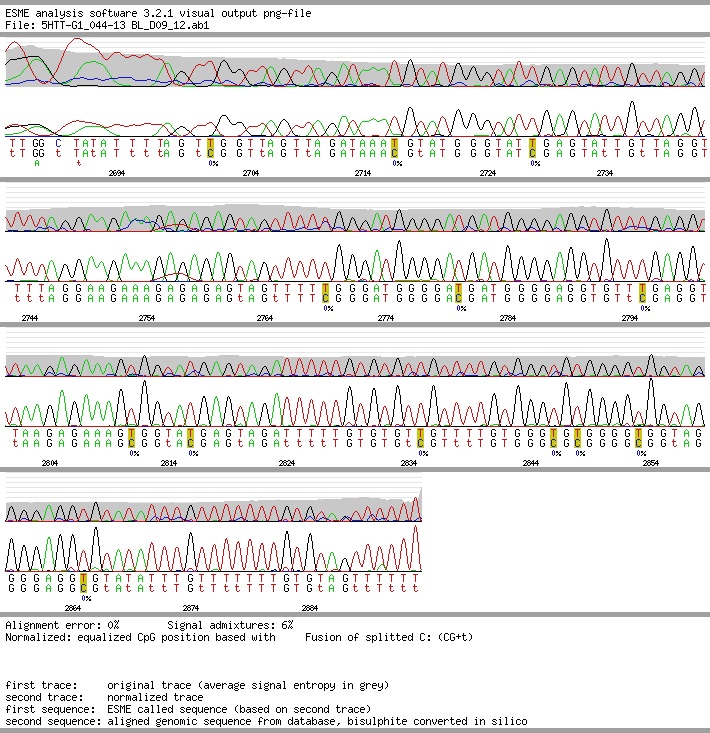

Supplement: Supplementary file 1 — Supplementary Material [file 41390_2021_1773_MOESM1_ESM.jpg]
